# Baroreceptor sensitivity, kidney function and cardiovascular risk in prepubescent boys with normal versus elevated blood pressure

**DOI:** 10.1038/s41371-025-01029-1

**Published:** 2025-05-28

**Authors:** Aletta S. Uys, Wayne Smith, Annemarie Wentzel, Catharina MC Mels, Ruan Kruger

**Affiliations:** 1 Hypertension in Africa Research Team (HART), North-West University, Potchefstroom, South Africa; 2 MRC Research Unit for Hypertension and Cardiovascular Disease, North-West University, Potchefstroom, South Africa

**Keywords:** Hypertension, Risk factors

## Abstract

In children reduced baroreceptor sensitivity (BRS) has been linked to obesity but not blood pressure (BP). Offspring of hypertensive parents have reduced BRS, with possibly increasing risk for hypertension development and kidney dysfunction. This study aimed to explore the relationships between BRS, kidney function, familial cardiovascular-and lifestyle risk in prepubescent boys with varying BP levels. We included 40 Black and 41 White boys (aged 6–8 years). Anthropometric measurements included calculated body mass index (BMI) and sex-and-age specific BMI z-scores (BMIz). Demographic data was collected with questionnaires and included information on familial cardiovascular-and lifestyle risk. Cardiovascular measures were resting BP and Finometer monitoring for BRS calculation. Kidney function was assessed using urinary albumin-to-creatinine ratio (uACR). Stratification was based on normal or elevated BP status. The elevated BP group had more Black boys (*n* = 37; 65.5%; *p* = 0.003). Notably, BRS (*p* = 0.56) and uACR (*p* = 0.92) were comparable between normal and elevated BP groups. In the normal BP group, single, partial and fully adjusted models revealed an inverse association between BRS and uACR (β = −0.38; *p* = 0.009). In the elevated BP group, BRS associated with familial risk (β = −0.52; *p* = 0.002), BMIz (β = 0.36; *p* = 0.020) and Black ethnicity (β = −0.37; *p* = 0.024), yet no association was evident between BRS and uACR. A cardioprotective relationship exists between BRS and kidney function in boys with normal BP. In boys with elevated BP, a positive familial cardiovascular-and lifestyle risk, adiposity and Black ethnicity seems to contribute to cardiovascular disease risk via a relationship with lower BRS.

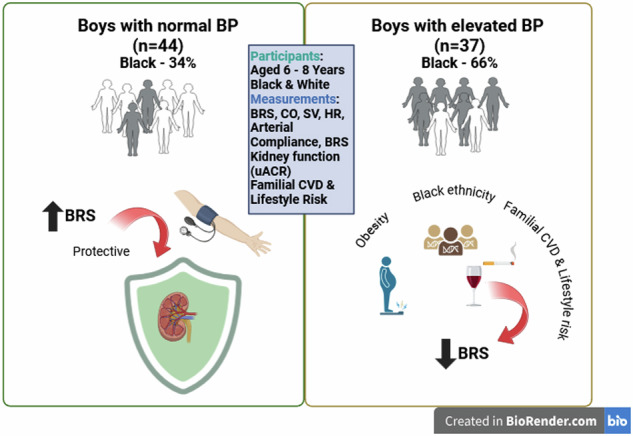

## Introduction

The baroreceptor reflex is one of the key autonomic regulators of blood pressure [1], and a significant decline in baroreceptor reflex sensitivity (BRS) is associated with sustained elevated blood pressure [2]. In fact, decreased BRS is implicated as a potential cause of hypertension, preceding increases in blood pressure (BP) rather than merely being a consequence thereof [3].

The prevalence of childhood hypertension is rapidly increasing worldwide, including in South Africa, with one in five children presenting with elevated blood pressure, and more than half of these cases persisting into adulthood [4]. Whether reduced BRS is already evident with elevated blood pressure during childhood is unknown as studies investigating BRS in paediatric cohorts are limited. One study in pre-adolescents reported reduced BRS linked to obesity and age but not blood pressure per se [5]. In obese children, reduced postural blood pressure adaptation has also implied baroreceptor dysfunction [6]. Another study determined that the offspring of hypertensive parents already presented with reduced BRS [7], possibly indicating a predisposition for developing hypertension in the future.

In children with high metabolic risk, increased sympathetic activity was associated with microalbuminuria [6]. This shift in autonomic balance in favour of the sympathetic nervous system, may lead to renal hyperfiltration, suggesting that microalbuminuria could serve as a marker of an autonomic imbalance [6]. If elevated blood pressure in childhood is accompanied by the early onset of chronic kidney disease [8, 9] and an imbalance in autonomic tone, the progression of cardiovascular disease (CVD) may be significantly augmented [10]. Adult patients with heart failure and chronic kidney disease, with impaired BRS, showed a worse cardiovascular mortality [11]. Although this finding pertains to a severely diseased patient population, it underscores the vital role of the baroreceptor reflex in regulating blood pressure and preventing CVD related organ damage possibly tracking into adulthood [12].

Thus, from adult based studies hypertension, imbalances in autonomic tone and kidney dysfunction/damage are interconnected, with each showing an interdependent relationship [13]. This interrelationship may already be evident in children as well, but data on these factors in paediatric populations are scarce. When considering the potential interplay between hypertension, imbalanced autonomic tone and kidney damage in children, it is important to consider other factors that have been identified as contributors linking to childhood hypertension such as obesity [14] and family history of CVD [15]. Therefore, the aim of our study was to determine whether BRS relates to kidney function as well as familial cardiovascular-and lifestyle risk factors in prepubescent boys stratified by blood pressure status.

## Methods

### Study design and population

The Arterial Stiffness in Offspring (ASOS) study was a cross-sectional observational study conducted in the city of Potchefstroom, North-West Province, South Africa. Approval for the study was obtained from the Provincial Department of Education as well as Health Research Ethics Committee of the North-West University (NWU-00007-12-A1). The participant group for this study consisted of 40 Black and 41 White healthy boys, aged between 6 and 8 years. Exclusion was based on use of self-reported chronic medication, self-reported type 1 diabetes mellitus, renal disease, or cancer.

Recruitment letters were circulated in schools to each child within the age range. Parents were invited to attend an information session at the school, hereafter received documents with information regarding the study and after a period of two weeks contacted to determine interest for their child’s participation. Before any measurements were taken, all procedures were explained to parents and children. Informed consent/assent were obtained from parents as well as the children to participate and relevant forms were completed and signed. Measurements were done in a 3-month period at the schools of the participants on Mondays – Fridays. The children were supplied with refreshments on the day of participation and detailed feedback on their basic health information after conclusion of the study.

### Questionnaire

Parents were supplied with a standard General Health Questionnaire to complete the day before the child’s participation and were requested to send it along with the child to school the next day. This questionnaire captured parental information regarding self-reported hypertension, history of stroke, dyslipidaemia, diabetes mellitus, prior myocardial infarction and/or coronary heart disease. Parental lifestyle risk factors included self-reported smoking and alcohol use. A composite familial cardiovascular susceptibility variable was also calculated based on parental cardiovascular and lifestyle risk factors. If a positive answer was indicated to any of the above cardiovascular or lifestyle risk factors in the questionnaire the composite cardiovascular susceptibility risk received a positive score (as indication of risk).

### Anthropometric measurements

Within a private room, the child’s body height (Invicta, Stadiometer, IP 1465, UK) and weight (Precision Health Scale, A & D Compony, Japan) were measured. Body mass index (BMI) was calculated as well as sex-specific BMI for age z-scores (BMIz) [16].

### Cardiovascular measurements

In a sitting position, resting BP measurements (systolic and diastolic) as well as heart rate were obtained in triplicate with a validated paediatric Omron HEM-759-E (750IT) device (Omron Healthcare, Tokyo, Japan) on the left upper arm while supported at heart level. Prior to BP measurement, participants were required to rest for at least 5 min. Hereafter, continuous resting cardiovascular measurements including mean arterial pressure (MAP), heart rate, arterial compliance, total peripheral resistance and BRS were obtained with the Finometer device (Finapres Medical Systems (FMS), Amsterdam, The Netherlands) [17]. Continuous BP measurements were done with the child lying down in a semi-upright position. The Beatscope v1.1 software (FMS, Amsterdam, The Netherlands) was employed to process Finometer data. Baroreceptor reflex sensitivity was calculated offline with the help of a dedicated program also developed by FMS (Amsterdam, The Netherlands). The program uses a cross-correlation method to calculate the BRS. It computes the correlation between beat-to-beat systolic blood pressure and inter-beat interval in a sliding 10 second window, with delays of 0 to 5 seconds per interval [18].

### Urine sampling and analysis

Children who chose to participate were supplied with a sealable urine cup the day before cardiovascular measurements were conducted. In the privacy of their own home, they were requested to supply a first voided midstream urine sample on the day of participation before any meal or fluids were taken. Children brought their urine samples along to school in a supplied cooler bag on the day of their participation along with the completed questionnaires. Urine samples were hereafter aliquoted and stored at −80 °C for planned biochemical analyses. Urinary albumin (intra-assay 1.9%; interassay 2.2%) and creatinine (intra-assay 1.4%; interassay 2.5%) were measured with the Cobas Integra 400 plus (Roche Diagnostics; Mannheim, Germany). The ratio between urinary albumin and creatinine was calculated (uACR).

### Statistical analysis

Statistical analyses were performed with IBM^®^ SPSS^®^ Statistics, Version 29 (IBM Corporation; Armonk, New York, USA). Variable normality was determined through visual inspection of QQ plots as well as observing skewness and kurtosis coefficients. In case of non-Gaussian distribution, logarithmic transformation was performed (uACR). Stratification in normal and elevated BP was done based on 2017 American Academy of Paediatrics Clinical Practice Guidelines [19] which states that participants BP levels within the 90^th^ percentile of their total group, are deemed as elevated. In a priori power analysis, using the G*power v3.1.9.3 software, a sample size of *N* was computed as a function of the required power level. The preselected power was 80% with the prescribed significance level estimated at α = 0.05. The population effect size was also detected at the probability of 1 - β (in this case 0.6) for the main outcome measure of the original study. The priori analysis calculated that an *N* value or population of 72 would be sufficient for the hypothesis of this study. Our study included 81 participants with 44 boys with normal blood pressure in one group and 37 boys with elevated blood pressure in the other group. Means and proportions were determined for the above-mentioned groups using independent *t* tests and chi-square tests respectively. Cardiovascular measures and uACR was hereafter compared with Analysis of Covariance, adjusting for BMIz and race. Pearson and partial correlations were performed to determine possible correlations between with BRS and familial cardiovascular composite risk variable, and uACR. Backward multiple linear regression analyses were performed to determine the relationships of BRS with familial cardiovascular and lifestyle risk as well as uACR whilst considering ethnicity, BMIz and age to determine a parsimonious model retaining only the most significant variables associated with BRS. A sensitivity analysis was performed where cardiovascular and lifestyle risk factors were separately considered. The regression models demonstrated homoscedasticity, and the residuals were approximately normally distributed.

## Results

Characteristics of the sample are shown in Table 1. The elevated BP group consisted of more Black boys (*p* = 0.03), displayed higher mean BMIz (*p* = 0.003) and BP values (all *p* < 0.001) compared to the normotensive group. Baroreceptor sensitivity (*p* = 0.56), heart rate (*p* = 0.64), stroke volume (*p* = 0.47), arterial compliance (*p* = 0.73), cardiac output (*p* = 0.27) and uACR (*p* = 0.92) values were all comparable between the two groups. After adjusting for race and BMIz as shown in Table 2 only resting heart rate (*p* = 0.01) was indicated as significantly higher in the boys with elevated blood pressure. It should however be noted that the effect size of the above-mentioned comparisons was small (Cohen’s d = 0.14).

**Table 1:** Characteristics of boys with normal and elevated blood pressure.

	Normal BP, *n* = 44	Elevated BP, *n* = 37	*P* value
Age (years)	7.7 ± 0.91	7.8 ± 0.79	0.62
Race, [Black, *n* (%)]	15 (34.1)	25 (65.6)	**0.003**
*Anthropometry*			
Height (cm)	125.99 ± 6.91	125.84 ± 6.60	0.92
Weight (kg)	25.12 ± 4.68	26.93 ± 5.21	0.10
BMI (kg/m^2^)	15.69 ± 1.49	16.89 ± 2.41	**0.004**
BMIz	−0.19 ± 0.93	0.41 ± 0.87	**0.003**
Waist circumference (cm)	56.46 ± 4.87	58.73 ± 6.42	0.08
*Cardiovascular profile*			
Systolic blood pressure (mmHg)	98.23 ± 6.46	110.78 ± 7.27	**<0.001**
Diastolic blood pressure (mmHg)	60.50 ± 6.61	72.73 ± 6.53	**<0.001**
Heart rate (bpm)	81.98 ± 9.41	82.97 ± 9.72	0.64
Mean arterial pressure (mmHg)	76.04 ± 5.50	88.42 ± 5.63	**<0.001**
Arterial compliance (ml/mmHg)	0.85 ± 0.73	0.91 ± 0.76	0.73
Cardiac output (l/min)	2.42 ± 2.01	3.01 ± 2.67	0.27
Stroke volume (ml/beat)	28.66 ± 23.56	32.70 ± 26.53	0.47
Baroreceptor sensitivity (ms/mmHg)	17.36 ± 7.17	16.33 ± 7.72	0.56
Family history of lifestyle and CVD risk, [*n* (%)]	26 (59.1)	13 (35.1)	0.07
*Kidney function*			
Albumin-to-creatinine ratio (mg/mmol)	0.63 (0.35–1.51)	0.63 (0.31–1.58)	0.92

Values are arithmetic mean ± SD, geometric mean (5^th^ and 95th percentile) or number of participants.

*BMI* body mass index, *BMIz* body mass index z-score, *CVD* cardiovascular disease.

**Table 2:** Adjusted comparison of cardiovascular measures and kidney function marker in blood pressure stratified groups.

	Normal BP, *n* = 44	Elevated BP, *n* = 37	*P* value
*Cardiovascular measures*			
Heart rate (bpm)	84.76 (81.95; 87.57)	90.50 (87.41; 93.59)	**0.01**
Arterial compliance (ml/mmHg)	0.83 (0.59; 1.06)	0.94 (0.68; 1.20)	0.54
Cardiac output (l/min)	2.31 (1.57; 3.04)	3.15 (2.34; 3.95)	0.14
Stroke volume (ml/beat)	27.52 (19.72; 35.32)	34.06 (25.47; 42.65)	0.29
Baroreceptor sensitivity (ms/mmHg)	17.77 (15.37; 20.17)	15.8 (13.22; 18.47)	0.31
*Kidney function*			
Albumin-to-creatinine ratio (mg/mmol)	0.63 (0.54; 0.73)	0.63 (0.53; 0.74)	0.92

Values are represented as adjusted mean with 95% Confidence Intervals. Variables are adjusted for Body Mass Index z-score and race.

Single regression analyses were performed within the groups (Table 3) revealing a significant association of BRS with age only in the elevated BP group (r = 0.40; *p* = 0.01). Age was therefore considered as confounding factor in further analyses along with ethnicity. In the normal BP group, BRS associated with uACR (r = −0.43; *p* = 0.006) in both single and partial regression analysis (r = −0.41; *p* = 0.01). Whilst in the elevated BP group, higher composite CVD risk was associated with attenuation of BRS in both single (r = −0.39; *p* = 0.02) and partial regression analysis (r = −0.55; *p* = 0.001).

**Table 3:** Pearson & Partial correlations in participant group stratified according to blood pressure.

	Normal BP, *n* = 44	Elevated BP, *n* = 37
*Pearson correlation with BRS (ms/mmHg)*		
Age (years)	***r*** = **0.40;** ***P*** = **0.01**	*r* = −0.12; *P* = 0.49
BMIz	*r* = 0.22; *P* = 0.17	*r* = 0.32*; P* = *0.06*
Waist circumference (cm)	***r*** = **0.38;** ***P*** = **0.02**	***r*** = **0.37;** ***P*** = **0.03**
Albumin-to-creatinine ratio (mg/mmol)	***r*** = −**0.43;** ***P*** = **0.006**	*r* = 0.19; *P* = 0.30
Family history of lifestyle and CVD risk	*r* = 0.27; *P* = 0.09	***r*** = −**0.39;** ***P*** = **0.02**
*Partial correlation with BRS (ms/mmHg)*^a^		
Albumin-to-creatinine ratio (mg/mmol)	***r*** = −**0.41;** ***P*** = **0.01**	*r* = 0.10; *P* = 0.61
Family history of lifestyle and CVD risk	*r* = 0.21; *P* = 0.22	***r*** = −**0.55;** ***P*** = **0.001**

^a^Adjusted for ethnicity, age, waist circumference.

In the normal BP group, multiple regression analysis revealed that BRS was inversely associated with uACR and positively with age (std. β = −0.38; *p* = 0.009) (Table 4). In the elevated BP group, multiple linear regression analysis revealed that a positive composite CVD risk (std. β = −0.52; *p* = 0.002) and higher BMIz (std. β = −0.36; *p* = 0.020) were associated with lower BRS while Black ethnicity (Std β = −0.54; *p* = 0.001) was also associated with lower BRS (std. β = −0.37; *p* = 0.024). In sensitivity analysis we also explored the contribution of CVD and lifestyle risk separately, but these variables did not enter the models.

**Table 4:** Independent relationships with baroreceptor sensitivity (BRS) within blood pressure stratified groups.

	Normal BP, *n* = 44		Elevated BP, *n* = 37	
*R*^2^	0.31		0.38	
Adjusted R^2^	0.27		0.31	
		*P* value		*P* value
Std β (95% confidence interval)				
Albumin-to-creatinine ratio (mg/mmol)	−0.38 (−0.98 to −0.15)	0.009	–	–
Age (years)	0.35 (1.20 – 10.34)	0.015	–	–
Family history of lifestyle and CVD risk	–	–	−0.52 (−1.11 to −0.26)	0.002
BMIz	–	–	−0.36 (0.07 to 0.79)	0.020
Ethnicity	–	–	−0.37 (−1.53 to −0.117)	0.024

Backward stepwise regression, variables considered for entering the model: age, ethnicity, body mass index z-score (BMIz), family history of lifestyle and CVD risk, albumin-to-creatinine ratio.

## Discussion

The aim of our study was to investigate the relationship between BRS (as an autonomic marker of blood pressure regulation) and kidney function in preadolescent boys with and without elevated blood pressure, while considering markers of adiposity and familial cardiovascular-and lifestyle risk. In boys with normal blood pressure, we found a protective association between BRS and uACR, but in boys with elevated blood pressure, BRS was associated with BMI and a positive family history of cardiovascular-and lifestyle risk.

In the early phases of primary hypertension, a hyperkinetic state is often indicated [20]. Increased sympathetic activity leads to an elevated heart rate and subsequently augmented stroke volume and cardiac output [20]. Our findings showed BRS, and other cardiovascular measures were comparable between boys with normal and elevated blood pressure. After adjusting for BMIz and race, the resting heart rate of the boys with elevated blood pressure were significantly higher, yet BRS, stroke volume and cardiac output remained comparable. A possible explanation for this finding is that our study sample consisted of young, healthy school children with blood pressure reported as a single measure at one time-point. Delimitation was based on elevated BP indicated when participants are stratified within the 90^th^ percentile and not necessarily hypertensive [19]. Therefore, in the boys with elevated blood pressure, pertinent autonomic imbalance in favour of sympathetic activity may not be evident yet, to a degree where a notable BRS difference could be seen. Despite the absence of significant observed differences in BRS and other cardiovascular measures, we persisted with further analysis based on the assumption that in the presence of elevated blood pressure an altered relationship between baroreceptor sensitivity and kidney function may already be indicated.

A shift in autonomic tone in favour of sympathetic activity, also pertains to kidney function in children. Rutigliano et al. suggested microalbuminuria as marker of autonomic imbalance based on glomerular hyperfiltration associating with sympathetic hyperactivity in a paediatric cohort [6]. Our findings in boys with normal blood pressure indicated a negative association between BRS and uACR possibly indicating the effective regulation of cardiovascular and renal function by the autonomic nervous system. Therefore, through efficient baroreflex control, blood pressure is adequately regulated, and renal damage is prevented preventing subsequent urinary leakage of albumin. Conversely so, the absence of this finding in boys with elevated blood pressure may hypothetically be an indication of the possible impairment of adequate blood pressure and kidney function modulation by the autonomic nervous system.

It is important to consider established risk such as adiposity and familial history, significantly contributing to childhood hypertension and possibly accompanying autonomic imbalance and inevitably affecting kidney function. Increased adiposity in children may negatively affect autonomic balance, shifting in favour of sympathetic activity and therefore adjusting blood pressure control [21]. Rutigliano et al. found altered autonomic function in children with high metabolic risk (obese or type 1 diabetic) [6]. Particularly in obese participants, greater blood pressure variability with postural change pointed to early BRS dysfunction. A study within this cohort by Mokwatsi et al. showed low incidence of obesity [22]. Irrespective of this, in our study the BMIz-score independently and inversely associated with BRS in the group with elevated blood pressure. This may indicate that with increase in body mass, an early shift in autonomic nervous system balance may occur, favouring increased sympathetic tone and modulation. This observation is additionally supported by the higher resting heart rate observed in this group.

Another factor that warrants consideration is the heritability of CVD risk factors whilst also considering parental lifestyle risk. The role of family history in contributing to CVD risk is underscored by our findings, which align with those of Mathews et al. [7] who reported that lower baroreflex sensitivity (BRS) associated with a familial predisposition to CVD. This may be due to epigenetic factors [12], environmentally influenced and eventually inherited mechanisms, which can amplify susceptibility to CVD by altering gene expression patterns associated with autonomic and cardiovascular regulation. These combined effects may have significant implications for blood pressure regulation and kidney function, potentially increasing susceptibility to CVD-related complications later in life.

Furthermore, our findings are also in alignment with previous studies who postulated earlier onset of vascular changes and affected blood pressure in Black paediatric cohorts [23]. This was however not the focus of our study but remains a significant factor to consider when evaluating early changes in autonomic regulation, blood pressure and hence kidney function in a South African context.

Consequently, the findings of our study emphasize in children the importance of considering autonomic regulation, adiposity, race and familial risk factors to possibly mitigate early shifts in autonomic balance and reduce long-term risks of hypertension, kidney dysfunction, and cardiovascular complications possibly later tracking into adulthood.

It is important to consider findings in the light of the studies’ strengths and limitations. A strength of this study is the inclusion of healthy young boys which allowed assessment of early life markers of cardiovascular and renal function. Furthermore, by including only boys this study tried to control for sex-based physiological differences. A limitation of this study was the cross-sectional observational design which prevents generalization to the broader population and no definitive cause-and-effect conclusion can be drawn. Furthermore, the classification of blood pressure was based on a once-off visit only. Hypertension diagnoses in paediatric cohorts can only be verified as guidelines specify with BP measured at three different time-points [19]. The authors also acknowledge the limitation of not considering dietary data and physical activity in the statistical models as these parameters were not included in the data collection of the main study. Another limitation to be noted is the reliance of a first-morning voided urine sample to determine uACR. We acknowledge that a 24-h urine collection would have been a better representation of daily urinary albumin and creatinine excretion, however, the implementation thereof in a paediatric cohort poses a logistic and compliance challenge. Result interpretation must be with caution due to the relatively small groups observed. Therefore, the small effect size could limit the magnitude of physiological interpretation.

## Conclusion

To conclude, we observed a cardioprotective relationship between BRS and kidney function in boys with normal blood pressure. A positive familial and lifestyle cardiovascular risk, Black ethnicity and adiposity seems to amplify cardiovascular disease risk by lower BRS in boys with elevated blood pressure. Our findings underscore the importance of early cardiovascular screening, relating to autonomic tone, and targeted interventions for at-risk youth to prevent long-term cardiovascular complications tracking into adulthood.

## Summary

### What is known about topic

- Adult based studies established the interconnection between autonomic imbalance, hypertension and kidney damage.
- In children, familial cardiovascular disease risk, obesity and autonomic imbalance alongside elevated blood pressure increase risk for kidney damage.

### What this study adds

- Our study confirmed a cardioprotective relationship between baroreceptor sensitivity and kidney function in a paediatric cohort of young, healthy boys with normal blood pressure
- In our cohort of boys with elevated blood pressure, familial cardiovascular and lifestyle risk as well as adiposity and Black ethnicity may increase cardiovascular disease risk through lower baroreceptor sensitivity.

## Data Availability

The datasets generated during and/or analysed during the current study are available from the corresponding author on reasonable request.
